# Phospholipase D1-regulated autophagy supplies free fatty acids to counter nutrient stress in cancer cells

**DOI:** 10.1038/cddis.2016.355

**Published:** 2016-11-03

**Authors:** Ming Cai, Jingquan He, Jian Xiong, Li Wei Rachel Tay, Ziqing Wang, Colin Rog, Jingshu Wang, Yizhao Xie, Guobin Wang, Yoshiko Banno, Feng Li, Michael Zhu, Guangwei Du

**Affiliations:** 1 Department of Gastrointestinal Surgery, Union Hospital, Tongji Medical College, Huazhong University of Science and Technology, Wuhan 430022, Hubei Province, China; 2 Department of Integrative Biology and Pharmacology, University of Texas Health Science Center at Houston, Houston, TX 77030, USA; 3 Department of Dermatology, Gifu University Graduate School of Medicine, Yanagido 1-1, Gifu 501-1194, Japan; 4 Department of Molecular and Cellular Biology, Baylor College of Medicine, Houston, TX 77030, USA

## Abstract

Cancer cells utilize flexible metabolic programs to maintain viability and proliferation under stress conditions including nutrient deprivation. Here we report that phospholipase D1 (PLD1) participates in the regulation of metabolic plasticity in cancer cells. PLD1 activity is required for cancer cell survival during prolonged glucose deprivation. Blocking PLD1 sensitizes cancer cells to glycolysis inhibition by 2-deoxy-D-glucose (2-DG) and results in decreased autophagic flux, enlarged lysosomes, and increased lysosomal pH. Mechanistically, PLD1-regulated autophagy hydrolyzes bulk membrane phospholipids to supply fatty acids (FAs) for oxidation in mitochondria. In low glucose cultures, the blockade of fatty acid oxidation (FAO) by PLD1 inhibition suppresses adenosine triphosphate (ATP) production and increases reactive oxygen species (ROS), leading to cancer cell death. In summary, our findings reveal a novel role of PLD1 in sustaining cancer cell survival during metabolic stress, and suggest PLD1 as a potential target for anticancer metabolism therapy.

One hallmark of cancer cells is increased aerobic glycolysis, widely known as the Warburg effect, that maintains cell growth and proliferation through the generation of adenosine triphosphate (ATP) and precursors for macromolecular synthesis.^1, 2^ However, although the Warburg effect empowers cells to proliferate rapidly when glucose is abundant, a flexible and alternative metabolic program must also be available for cancer cells to respond readily to conditions leading to metabolic stresses, for example, rapid tumor growth and exposure to therapy.^2, 3^ This metabolic plasticity explains why, in theory, therapies aimed at inhibiting glucose utilization in ‘glucose-addicted' cancer cells should be efficacious at eliminating tumors, but in practice, cancer cells often resist to approaches that target glycolysis alone.^2, 3^ One major mechanism by which cancer cells adapt to nutrient scarcity is to shift their dependence on glycolysis to mitochondrial fatty acid oxidation (FAO) that serves to sustain ATP levels and counter oxidative stress.^4, 5, 6^ The biochemical basis for this metabolic reprogramming under metabolic stress is largely known.^4, 5^ However, how fatty acids (FAs) are generated, mobilized, and transferred into mitochondria for subsequent utilization remains unclear.

Autophagy is a tightly regulated process that maintains cellular homeostasis by lysosomal processing of damaged cellular organelles and long-lived proteins. It is generally accepted that autophagy suppresses tumor initiation at the early stages of cancer but promotes tumor growth by providing a source of nutrients during rapid tumor growth.^7, 8^ In fact, in many tumor cells, autophagy activation is required for proliferation and survival, as well as the development of resistance to the treatment.^7, 8^ Therefore, inhibiting autophagy may be beneficial for cancer therapy. One key mechanism by which autophagy promotes tumorigenesis is through the regulation of lipid metabolism.^6, 9^ However, despite the rapid progress in autophagy research, the underlying mechanisms by which autophagy promotes and maintains the growth of tumors remain undefined.

In this paper, we report that the activity of phospholipase D1 (PLD1) is required for metabolic reprogramming of cancer cells enduring prolonged glucose deprivation. PLD1 inhibition blocks autophagic flux and free FA production from bulk membrane phospholipids that in turn limits FAO in mitochondria. This results in a decrease in cytosolic ATP level and an increase in mitochondrial reactive oxygen species (ROS) production, promoting cancer cell death during glucose deprivation.

## Results

### PLD1 is required for cancer cell survival during prolonged glucose deprivation

**Figure 1:**
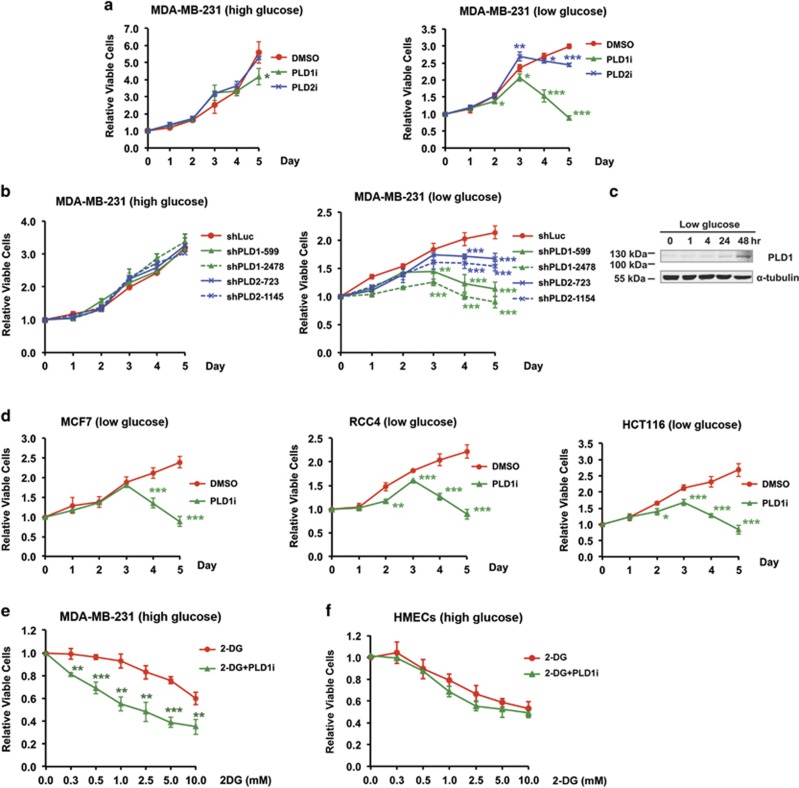
Requirement of PLD activity for cancer cell survival in low glucose medium. (**a**) PLD1 and PLD2 small-molecule inhibitors, PLD1i and PLD2i, reduced the viability of MDA-MB-231 cells when the culture medium contained low glucose (1 mM) but not when it had high glucose (25 mM); *n*=3. (**b**) shRNA knockdown of PLD1 and PLD2 in MDA-MB-231 cells reduced cell viability in low glucose but not high glucose medium. Two shRNA constructs were used for each PLD isoform; *n*=3. Statistical significances were labeled on days 3–5. (**c**) Glucose deprivation increased the expression of PLD1 protein after 24  h. The experiments were repeated twice with similar result. (**d**) PLD1i reduced the viability of MCF-7 (breast), RCC4 (renal), and HCT116 (colorectal) cancers cells grown in low glucose medium; *n*=3. (**e**) PLD1i sensitized MDA-MB-231 cells to cell death promoted by 2-DG. MDA-MB-231 cells grown in high glucose medium were treated with PLD1i (5 *μ*M) and the indicated concentrations of 2-DG. Cell viability was measured on day 3; *n*=3. (**f**) PLD1i did not have an addition effect on the viability of 2-DG-treated HMECs. HMECs cells grown in high glucose medium were treated with PLD1i (5 *μ*M) and the indicated concentrations of 2-DG. Cell viability was measured on day 3; *n*=3. *P<0.05; **P<0.01; ***P<0.001

When treated with isoform-specific PLD inhibitors, the viability of MDA-MB-231 breast cancer cells grown in the regular culture medium that contained high glucose (25 mM) was not significantly altered by either the PLD1 inhibitor (PLD1i) or PLD2 inhibitor (PLD2i) (Figure 1a). However, both inhibitors, especially PLD1i, caused significant decreases in cell viability as early as day 3 in low glucose (1 mM) medium (Figure 1a). Furthermore, PLD1 and PLD2 knockdown by small hairpin RNAs (shRNAs)^10, 11^ (Supplementary Figure S1) also reduced the viability of MDA-MB-231 cells starting at day 3 in low, but not high, glucose medium (Figure 1b). As both PLD1 shRNAs and PLD1i showed greater effects on cell viability than those of PLD2 (Figures 1a and b), we focused on PLD1 in subsequent studies. Interestingly, the expression of PLD1 protein in MDA-MB-231 cells was increased 2 days after exposure to low glucose (Figure 1c), further supporting a critical role of PLD1 in protecting the cancer cells from glucose starvation.

We then measured the effect of PLD1 inhibition on the viability of three other cancer cell lines, including MCF-7 (breast), RCC4 (renal), and HCT116 (colorectal). Although PLD1i caused some moderate reduction in viable MCF-7 and RCC4 cells in high glucose (Supplementary Figure S2), it robustly inhibited the growth of all three cell types starting from day 3 in low glucose (Figure 1d). Therefore, the requirement for PLD1 activity to counter glucose deprivation is common among cancer cells.

As PLD1 inhibition decreased cancer growth in low glucose medium, we reasoned that this treatment might also sensitize cancer cells to glycolysis inhibition. Therefore, we treated MDA-MB-231 cells grown in the high glucose medium with different concentrations of 2-DG, a glycolytic inhibitor, in the absence and presence of PLD1i. Co-application of PLD1i significantly reduced the numbers of viable cells at all tested 2-DG concentrations (Figure 1e). In contrast, although 2-DG itself reduced the proliferation of nontumorigenic human mammary epithelial cells (HMECs), addition of PLD1i did not have additional effect (Figure 1f). These data support the potential use of PLD1i in cancer combination therapy.

### PLD1 regulates autophagic flux independent of mTOR activity

**Figure 2:**
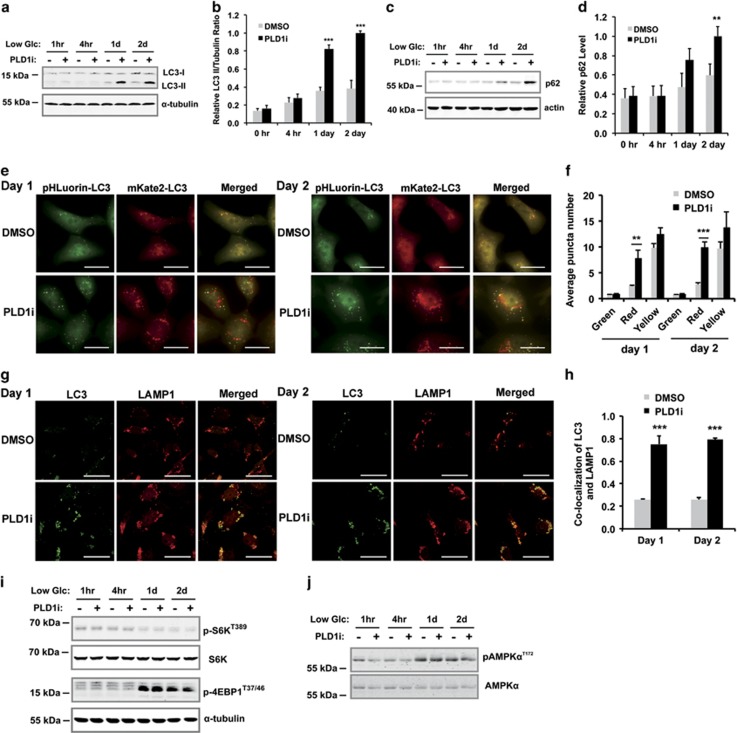
PLD1 inhibition reduces autophagic flux without affecting initiation. (**a**) PLD1i treatment increased the levels of LC3-II and p62 in MDA-MB-231 cells cultured in low glucose medium (1 mM). Cells were treated with or without PLD1i for the indicated time period. Total cell lysates were analyzed by western blotting for the indicated proteins. (**b**) Quantification of the ratio of LC3-II and the loading control, *α*-tubulin, using ImageJ; *n*=3. (**c**) PLD1i treatment increased the levels of p62 in MDA-MB-231 cells cultured in low glucose medium. Cells were treated with or without PLD1i for the indicated time period. Total cell lysates were analyzed by western blotting for the indicated proteins. (**d**) Quantification of p62 levels using ImageJ, and normalized to the loading control, actin; *n*=3. (**e**) MDA-MB-231 cells stably expressing pHluorin-mKate2-LC3 were cultured in low glucose medium and treated with DMSO (control) or PLD1i (5 *μ*M) for 1 (left) and 2 days (right) before images for pHluorin and mKate2 fluorescence were taken (scale bar: 20 *μ*m). (**f**) Quantification of green (pHluorin) and red (mKate2) and yellow (both pHluorin and mKate2) puncta per cell in merged pictures. At least 20 cells were quantified in each experiment; *n*=3. (**g**) MDA-MB-231 cells cultured in low glucose medium were treated with DMSO or PLD1i for 1 (left) and 2 days (right) before immunostaining performed for LC3 (green) and LAMP1 (red) (scale bar: 20 *μ*m). Cells were permeabilized with Triton X-100. (**h**) Quantification of colocalization between LC3 and LAMP1; *n*=3. (**i**) PLD1i treatment did not change mTOR activity as assessed by the phosphorylation of p70S6K and 4E-BP1. Note the low glucose culture increased phosphorylation of 4E-BP1 on day 1 and day 2, but PLD1i did not alter this effect. (**j**) PLD1i treatment did not change AMPK activity as assessed by the phosphorylation of AMPK*α*. **P<0.01; ***P<0.001

To understand the mechanisms through which PLD1 protects cells against metabolic stress, we examined the activity of autophagy, a common stress response pathway. The LC3-II level, a marker of autophagy, increased slightly after switching to low glucose by western blotting. In contrast, PLD1i treatment strongly and significantly increased LC3-II levels after 1 and 2 days (Figures 2a and b). The increased LC3-II levels could be a consequence of enhanced autophagy initiation or decreased autophagic flux.^12, 13^ To determine the cause for the increase in LC3-II, we measured the level of p62. Efficient flux of autophagy would result in a decrease in p62.^12^ PLD1i treatment resulted in an increase in p62 (Figures 2c and d), suggesting a blockage of autophagic flux that often signifies a failure of autolysosome function.^12^ We also measured the levels of LC3 and p62 in cells in which autophagy was blocked by shRNA knockdown of ATG5, an essential autophagy initiation protein.^13, 14^ ATG5 knockdown blocked the increased levels of LC3-II (Supplementary Figure S3), indicating the blockade of autophagy. ATG5 knockdown alone increased the levels of p62 as previously reported.^12^ However, combination of ATG5 knockdown and PLD1i did not further change the levels of p62 as compared with ATG5 knockdown alone (Supplementary Figure S3), suggesting that inhibition of autophagic degradation is the cause of p62 accumulation in PLD1i-treated cells, and is unlikely caused by the upregulation of transcription or inhibition of proteasomal degradation as reported in some stress conditions.^12^

We next stably expressed a tagged LC3 that fused to a tandem pHluorin-mKate2,^15^ an improved tool of the monomeric red fluorescent protein (mRFP) –green fluorescent protein (GFP)–LC3 (tfLC3).^16, 17^ Because the pHluorin (green), but not the mKate2 fluorescent signal (far-red fluorescence, pseudocolored as red), is quenched by the acidic environment of lysosomal lumen, the coexistence of both pHluorin and mKate2 fluorescent signals (yellow) indicates LC3-containing vesicles before fusion with lysosomes, whereas those showing red represent autolysosomes.^12, 17^ We found that PLD1i significantly increased the red signal in pHluorin-mKate2-LC3-expressing cells (Figures 2e and f), indicating an accumulation of LC3 in autolysosomes. In support of this, PLD1i treatment also increased the colocalization of LC3 with LAMP1, a lysosome marker (Figures 2g and h). Taken together, PLD1i likely affects the terminal step of autophagic flux, autolysosomal degradation. On the other hand, treatment of PLD1i in the first 2 days did not exert a detectable effect on the activity of mTORC1 (measured by 4E-BP1 and p70S6K phosphorylation) (Figure 2i), AMPK (measured by AMPK*α* phosphorylation) (Figure 2j), and other signaling pathways, including p38, extracellular signal-regulated kinase (ERK), or AKT (Supplementary Figure S4). These results also support that PLD1 is involved in autophagic flux but not autophagy initiation.

**Figure 3:**
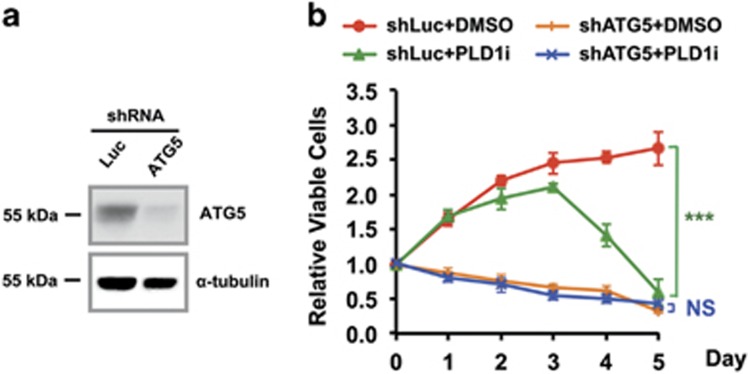
Autophagy is required for PLD1-regulated cell growth in low glucose medium. (**a**) Western blot of ATG5 knockdown by shRNA in MDA-MB-231 cells. (**b**) The knockdown of ATG5 by shRNA suppressed the growth of MDA-MB-231 cells right after culturing them in low glucose medium. PLD1i treatment did not aggravate the effect of autophagy inhibition by ATG5 shRNA. Luciferase shRNA (shLuc) was used as a negative control; *n*=3. ***P<0.001

To investigate whether autophagy is required for the survival of MDA-MB-231 cells in low glucose, we measured viability in ATG5 knockdown cells (Figures 3a and b). The loss of ATG5 alone led to gradual decreases in cell viability beginning 1 day in the low glucose medium (Figure 3b). The addition of PLD1i did not further increase cell death (Figure 3b). These data suggest that cancer cells use autophagy to maintain growth under glucose-starved conditions and PLD1 activity is critically involved in this pathway through supporting autophagic flux.

### PLD1 inhibition results in the perinuclear clustering, enlargement, and alkalinization of lysosomes

**Figure 4:**
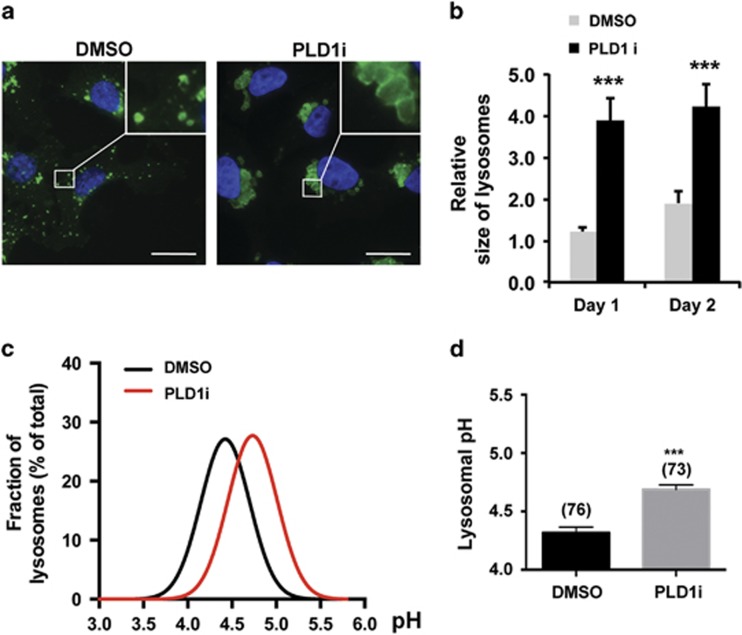
PLD1 inhibition impedes lysosomal functions. (**a**) PLD1 inhibition caused prominent lysosome enlargement. MDA-MB-231 cells were grown in low glucose medium and treated with DMSO or PLD1i for 2 days. Cells were fixed and then permeabilized with saponin that preserved good lysosomal morphology before staining for LAMP1 (green) and DAPI for nuclei (blue) (scale bar: 20 *μ*m). (**b**) Quantification of lysosomal size in MDA-MB-231 cells as in (**a**). Lysosome size was determined by the areas of LAMP1-labeled vesicles using ImageJ; *n*=3. (**c**) PLD1 inhibition increased lysosomal pH. Distribution histograms of lysosomal pH values (fitted to Gaussian distributions) measured by ratiometric imaging from DMSO and PLD1i-treated MDA-MB-231 cells grown for 2 days in low glucose medium. (**d**) Mean lysosomal pH values of data points in (**c**); *n*=3. ***P<0.001

As reported in other cell types,^18, 19^ a significant amount of HA-tagged PLD1 was found in intracellular puncta that are labeled by the lysosomal marker, LAMP1 (Supplementary Figure S5). This lysosomal localization remained largely unchanged by glucose starvation and PLD1i treatment (Supplementary Figure S5). However, PLD1i treatment led to enlargement of lysosomes and their clustering around nuclei (Figures 4a and b). These changes were more prominent in cells permeabilized with a weak detergent, saponin (Figures 4a and b), than those treated with Triton X-100 (Figures 2e and f and Supplementary Figure S5).

Macromolecule digestions in lysosomes are carried out by luminal hydrolytic enzymes that work optimally at acidic pH.^20^ We performed ratiometric lysosomal pH measurement in MDA-MB-231 cells loaded with pH-sensitive Oregon Green 488-dextran that exhibits pH-dependent changes in excitation and emission.^21, 22^ The PLD1i-treated cells displayed an elevated mean lysosomal pH value of 4.74 as compared with the control value of 4.30 obtained from untreated cells (Figures 4c and d). These observations suggest that inhibiting PLD1 disrupts lysosomal functions that may underlie the autophagy defect and reduced viability in MDA-MB-231 cells under glucose-deprived and PLD1-inhibited conditions.

### PLD1-regulated FAO sustains cancer cell survival during glucose deprivation

**Figure 5:**
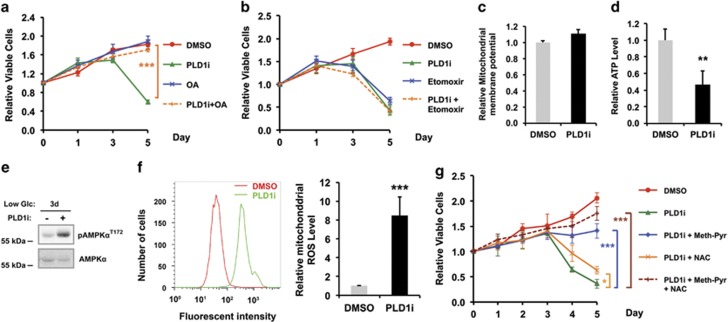
PLD1 regulates free FA generation to support mitochondrial FAO in MDA-MB-231 cells exposed to low glucose medium. (**a**) Addition of exogenous oleic acids fully rescued cell death induced by PLD1i; *n*=3. (**b**) Etomoxir, a FAO inhibitor, reduced viability of MDA-MB-231 cells similarly as PLD1i. The combined treatment with etomoxir and PLD1i had no additive effect; *n*=3. (**c**) PLD1i had no effect on mitochondrial membrane potential; *n*=3. (**d**) PLD1i reduced ATP production; *n*=3. (**e**) PLD1i treatment increased the phosphorylation of AMPK*α* on day 3 in low glucose medium. (**f**) PLD1i increased mitochondrial ROS level. The MDA-MB-231 cells stained with MitoSOX Red mitochondrial ROS indicator were analyzed by flow cytometry. Left, histograms of MitoSOX fluorescent intensity. Right, statistic results of the relative mitochondrial ROS level; *n*=3. (**g**) The cell death phenotype of PLD1i-treated cells was rescued by methyl-pyruvate (meth-Pyr) and *N*-acetylcysteine (NAC); *n*=3. *P<0.05; **P<0.01; ***P<0.001

As FA oxidation is critical to energy production and countering oxidative stress during metabolic stresses,^4, 5, 6^ the PLD1 inhibition-induced alteration of lysosomal functions might impair FA production needed for cancer cell survival during glucose deprivation. Indeed, supplementation of oleic acids, one of the abundant FAs in the cell, completely rescued cell death phenotype caused by PLD1i (Figure 5a). We next treated cells grown in low glucose with etomoxir, a FAO inhibitor. Similar to PLD1i, etomoxir caused a marked decrease in cell viability starting from day 3 in low glucose (Figure 5b). Co-application of PLD1i with etomoxir did not further reduce the viability (Figure 5b), indicating that the two inhibitors affect the same metabolic pathway. In addition, treatment of PLD1i had no effect on mitochondrial membrane potential, suggesting that PLD1 is not directly involved in mitochondrial function (Figure 5c).

We then measured intracellular ATP and ROS levels. PLD1i significantly decreased cytosolic ATP on day 3 of the low glucose culture when cell viability began to drop (Figure 5d). Consistent with ATP level, PLD1i treatment also significantly increased AMPK activity on day 3 of low glucose treatment (Figure 5e). PLD1i also caused a robust increase in mitochondrial ROS level (Figure 5f), whereas it had no effect on the cytosolic ROS level (Supplementary Figure S6), supporting the importance of PLD1 in facilitating mitochondrial FAO. Together with the unchanged mTOR and AMPK activities in PLD1i-treated cells on day 1 and 2 (Figures 2i and j), these data further support that PLD1 is not involved in the initiation of autophagy; however, PLD1-regulated autophagic degradation is responsible for fatty acid oxidation, ATP, and ROS production. Importantly, exogenous supplements of methyl-pyruvate, a membrane-permeable form of pyruvate that provides the substrate for ATP production from oxidative phosphorylation in mitochondria,^23^ greatly rescued the cells from PLD1i-induced death in low glucose (Figure 5g). *N*-acetylcysteine (NAC), an antioxidant, also had moderate rescue effect (Figure 5g). Co-application of both methyl-pyruvate and NAC with PLD1i further enhanced cell viability to the levels approaching the untreated cells (Figure 5g). Collectively, these findings suggest that PLD1 plays a critical role in FA production that supports FAO in mitochondria, therefore generating energy and antioxidative stress capacity needed for cancer cell survival under glucose-deprived conditions.

### PLD1 regulates free FA production from membrane phospholipids during prolonged glucose starvation

**Figure 6:**
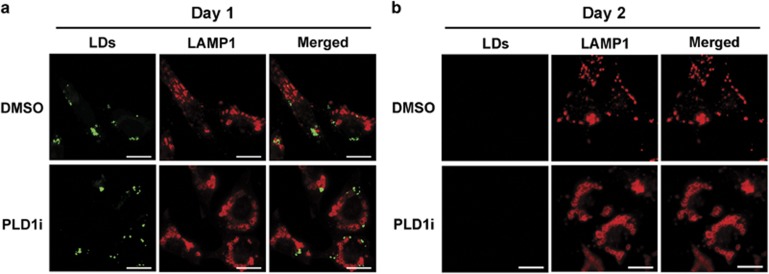
PLD1 is not involved in the homeostasis of lipid droplets. MDA-MB-231 cells grown in low glucose medium for 1 day (**a**) or 2 days (**b**) were stained with BODIPY493/503 for lipid droplets and LAMP1 for lysosomes (scale bar: 20 *μ*m). Cells were permeabilized with saponin

During nutrient starvation, free FAs can be released either from lipid droplets by lipase-mediated lipolysis or from autophagic digestion of membrane-bound organelles (e.g., the endoplasmic reticulum) or lipid droplets (by a form of autophagy called lipophagy).^24, 25^ We first examined whether PLD1 inhibition caused accumulation of lipid droplets. Lipid droplets were abundant in both control and PLD1i-treated cells on day 1 after exposure to low glucose (Figure 6a), consistent with the previous observations in short-term starvation.^26, 27, 28^ By day 2, the lipid droplets were barely detectable (Figure 6b), presumably because of depletion of stored lipids under the low glucose conditions. This result also suggests that lipids previously stored in lipid droplets contribute minimally to cell survival under long-term glucose starvation (beyond 3 days in our condition). PLD1 inhibition had no effect on the depletion of the lipid droplets (Figures 6a and b), suggesting that PLD1 is not involved in the utilization of lipid droplets during glucose starvation. Furthermore, PLD1 did not affect the colocalization between lipid droplets and LAMP1 (Figures 6a and b).

**Figure 7:**
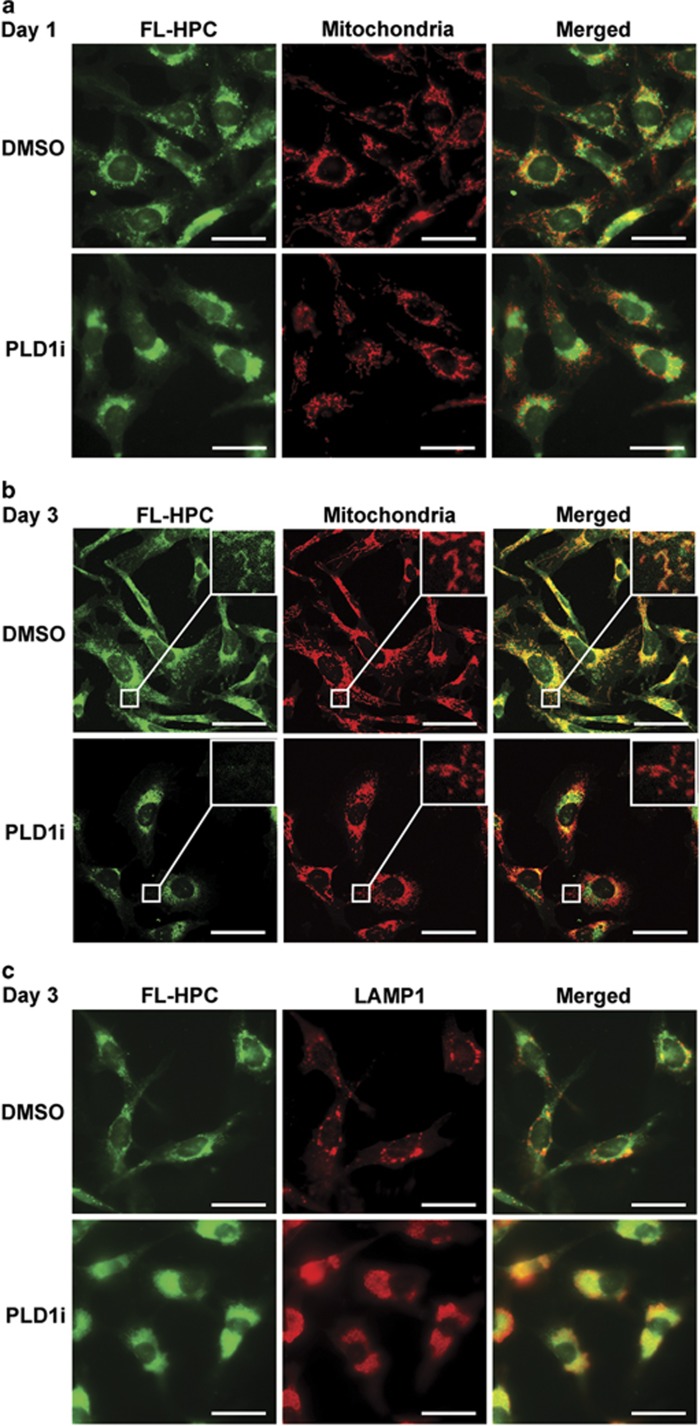
PLD1 regulates the mobilization of phospholipids from cellular membranes to mitochondria during glucose starvation. (**a** and **b**) MDA-MB-231 cells were labeled with FL-HPC overnight, and chased in low glucose medium for 1 day (**a**) or 3 days (**b**) in the presence or absence of PLD1i (scale bar: 20 *μ*m). Cells were permeabilized with saponin. Mitochondria were labeled by TOM22. (**c**) Inhibition of PLD1 led to the accumulation of FL-HPC in LAMP1-labeled autolysosomes on day 3 (scale bar: 20 *μ*m)

To determine the source of free FAs, we labeled membranes with a phosphatidylcholine species that contained a fluorescence tag at its FA tail (FL-HPC). Similar to fibroblasts,^28^ the green fluorescence of FL-HPC labeled various cellular membranes and only partially colocalized with mitochondria in MDA-MB-231 cells 1 day after labeling (Figure 7a), and remained largely unchanged on day 2. By day 3 when cells started to die in the low glucose medium (Figure 1), all mitochondria were strongly labeled with the green fluorescence, presumably by channeling free fatty acids hydrolyzed from FL-HPC into mitochondria as demonstrated before^28^ (Figure 7b). Although PLD1 inhibition did not seem to change the FL-HPC labeling pattern on day 1 (Figure 7a), it greatly reduced green fluorescence in the mitochondria (especially those in cell peripherals) on day 3 (Figure 7b), concurrent with an accumulation of the green fluorescence in autolysosomes (Figure 7c). Taken together, these results suggest that PLD1-regulated autophagy hydrolyzes membrane phospholipids rather than triglycerides in lipid droplets to supply FAs for oxidation in mitochondria.

## Discussion

### PLD1 regulation of autophagy and lysosome functions

PLD1 activity has been implicated in autophagy in several previous studies.^29, 30, 31, 32^ However, the mechanism and functional implication of PLD1 regulation of autophagy remain unclear. We found that PLD1 inhibition has no effect on mTOR activity, but rather it increases LC3 and p62 accumulation in autolysosomes, lysosomal size, and lysosomal pH (Figures 2 and 4), supporting that PLD1 functions in the late stages of autophagy, most likely autolysosomal degradation, during prolonged glucose starvation. Because an optimal acidic pH is critical to the activity of lysosomal enzymes,^20^ the increased pH may compromise digestion and be responsible for the detected accumulation of LC3, p62, and phospholipids. The failure of phospholipid hydrolysis reduces the production of free FAs that in turn limits FAO for ATP production and ROS reduction under glucose deprivation conditions. Therefore, our current study reveals a mechanism and the functional consequence of PLD1-regulated autophagy in long-term glucose starvation. It is worth noting that the involvement of PLD1 in lysosomal pH regulation does not necessarily rule out the previously proposed role for PLD1 in autophagosome–lysosome fusion^29, 31^ that can be compromised by an elevation in lysosomal pH.^33^

Similar to Vps34,^34^ PLD1 appears to regulate autophagy in a context-dependent manner. Depending on cell types and states, and experimental conditions, PLD1 may regulate both initiation and completion of autophagy, and function as both positive and negative regulator of autophagic flux.^30, 31, 32^ Jang *et al.*^30^ reported that PLD1 inhibition increases LC3-II levels, whereas it reduces p62 levels and promotes autophagic flux. Dall'Armi *et al.*^29^ reported that PLD1 inhibition reduces the LC3-II levels but increases p62 levels. Bae *et al.*^31^ and our current study showed that PLD1 inhibition increases the levels of both LC3-II and p62 as a result of inhibition of autophagic flux. It is unclear how PLD1 functions at the different steps of autophagy. One possibility is that different autophagy-induction conditions, for example, complete depletion of all nutrients^29, 30^ *versus* low glucose condition in our study, differently modulate the activities of PLD1 or other autophagy regulators at various steps in autophagy. It is also possible that the uses of different sources or concentrations of PLD1 inhibitors cause unexpected off-target effects.

A remaining question is how PLD1 regulates lysosomal pH. Lysosome pH is controlled primarily by the vacuolar-type H^+^ ATPase (V-ATPase) that translocates protons into lysosomal lumen and facilitated by transporters and channels that either promote the influx of cytosolic anions into the lysosomes or the efflux of luminal cations to cytosol to compensate for change in the transmembrane electrical potential generated by luminal proton accumulation.^20^ PLD1 generates the signaling lipid phosphatidic acid (PA). It has been known that phospholipids are important regulators of many channels.^35^ It is likely that PLD1-generated PA is involved in the regulation of the transportation of protons and/or counterions in lysosomes. How PA regulates the activities of lysosomal V-ATPase, transporters, and channels would be an important area to explore in the future.

### Generation of FAs from bulk membrane phospholipids during nutrient deprivation

Recent studies suggest that pleiotropic role of autophagy in lipid metabolism may be tissue or condition specific.^25, 28, 36^ Although lipolysis of lipid droplets is the major source of intracellular FAs in fibroblasts during acute starvation in Hanks' balanced salt solution (HBSS) that lacks glucose, amino acids, and growth factors,^28^ lipophagy plays a more critical role in conditions of serum depletion in the presence of amino acids and glucose.^25, 28, 36^ In the current study, lipolysis by cytosolic lipases and lipophagy of lipid droplets occurred within 2 days after exposure of the cancer cells to low glucose; therefore, they do not seem to play a significant role in PLD1-regulated cell viability that did not show an effect until 3 days after glucose starvation (Figures 1 and 6). In contrast, we demonstrated that PLD1-regulated autophagic flux supplies FAs to mitochondria from bulk membrane phospholipids in response to prolonged glucose starvation (Figure 7). Noticeably, the aforementioned studies^25, 28, 36^ focused on relatively short-term starvation (hours to 1 day), and/or performed experiments in an extreme condition, for example, HBSS. It remains to be addressed how different sources of FAs, that is, lipolysis, lipophagy, and autophagy of bulk membranes, contribute to different cellular functions, such as proliferation and viability, during early and late phases of starvation conditions lacking different forms of nutrients, for example, amino acids and glucose. One possibility is that different nutrient deprivation stresses activate overlapping yet distinct signaling pathways that determine the use of different mechanisms to obtain intracellular FAs. Another possibility is that regardless of the nutrient stress type, membrane phospholipids are the major substrates of FA production in the late phase after exhaustion of other sources. Although it has been speculated for some time that membrane phospholipids may supply free FAs to cells via autophagy,^8, 37^ no experimental evidence has appeared until the current study. More studies need to be done to further understand how autophagic degradation of membrane phospholipids contributes to cellular functions, for example, energy production, ROS reduction, and resynthesis of lipids.

### Targeting PLD1-regulated metabolic reprogramming in cancer therapy

A major obstacle of targeting cancer metabolism is the metabolic plasticity that allows cancer cells to adapt to harsh conditions for proliferation and/or survival, and developing resistance to metabolic cancer therapeutics.^2, 3, 6^ Our data show that during prolonged glucose deprivation, cancer cells rely on FAs recycled from membrane phospholipids for survival. Although FAs may be produced by lipolysis and lipophagy from preexisting lipid droplets, they do not seem to affect cell survival during prolonged glucose starvation. It has been previously shown that autophagy provides FAs to maintain growth and survival of a dormant population of tumor cells.^9, 38^ Our data demonstrate that during prolonged glucose starvation, membrane phospholipids are the source of autophagy-derived FAs that support ATP synthesis and suppress excess ROS production in mitochondria; and this process is regulated by PLD1 (Figures 5 and 7). Our data underscore the importance of recycling membrane phospholipids via autophagy to cancer cell survival during nutrient scarcity and establish PLD1 as a critical player in this important metabolic adaption pathway.

Our current study shows that inhibition autophagy by ATG5 knockout causes significant death of MDA-MB-231 cells in low glucose condition, and PLD1 inhibition is unable to further increase cell death in this condition (Figure 3). This result suggests that PLD1 regulates cell survival mainly through autophagy, and metabolic cancer therapeutics attacking the Warburg effect would be more effective when PLD1 or PLD1-regulated signaling pathways are inhibited. Our data also support a previous finding that MDA-MB-231 cells are very sensitive to autophagy inhibition.^39^ In contrast, it was proposed by Jang *et al.*^30^ that the combination of PLD1 and autophagy inhibition might be of benefit to cancer patients, based on the result that PLD1 inhibition reduced cell viability in ATG7 knockdown MDA-MB-231 cells. This result suggests that PLD1 regulates cell death mainly through an autophagy-independent role. It remains unclear whether this apparent discrepancy is caused by different glucose concentrations (1 mM in current study and 0 mM and in Jang *et al.*^30^, respectively), different degrees of autophagy inhibition by ATG5 (our study) and ATG7 knockdown (Jang *et al.*^30^), or different concentrations and sources of PLD1 inhibitors (5 *μ*M from Avanti Polar Lipids in current study and 10 *μ*M from Cayman Chemical in Jang *et al.*^30^, respectively). Further clarifying the mechanism through which PLD1 inhibition results in cancer cell death would provide a better cancer therapy.

## Materials and Methods

### General reagents and antibodies

PLD1i (VU0359595) and PLD2i (VU0285655-1) were from Avanti Polar Lipids (Alabaster, AL, USA). Antibodies for phosphorylated and/or total AKT (1 : 1000), p70S6K (1 : 1000), 4E-BP1 (1 : 1000), p38 (1 : 1000), ATG5 (1 : 1000), and LC3 (1 : 1000) were from Cell Signaling Technology (Danvers, MA, USA). Mouse monoclonal p62 antibody was from BD Biosciences (San Jose, CA, USA). FIPI, antibodies for *α*-tubulin (1 : 5000), phospho-ERK1/2 (1 : 40 000), and total ERK1/2 (1 : 40 000) were from Sigma-Aldrich (St. Louis, MO, USA). LAMP1 was from the Developmental Studies Hybridoma Bank (Iowa City, IA, USA). Rabbit monoclonal PLD1 antibody (1 : 1000) was from Abcam (Cambridge, MA, USA). Rabbit polyclonal PLD2 antibody (1 : 500) has been described before.^40, 41^ Goat anti-mouse and anti-rabbit IgGs conjugated with Alexa Fluor 488 or 594 were from Life Technologies (Grand Island, NY, USA). Goat anti-mouse and anti-rabbit IgGs conjugated with IRDye 680CW or IRDye 800CW (1 : 5000) were from Rockland Immunochemicals (Gilbertsville, PA, USA).

### Cell culture and viability assays

All cancer cells were maintained in Dulbecco's modified Eagle's medium (DMEM) supplemented with 10 % fetal bovine serum (Life Technologies). The immortalized nontumorigenic HMECs were cultured in 1/2 MEBM Mammary Epithelial Cell Growth Medium from Lonza (Allendale, NJ, USA) and 1/2 DMEM/F12 supplemented with insulin (5 *μ*g/ml), EGF (10 ng/ml), and hydrocortisone (0.5 *μ*g/ml) as described before.^42^ For cell viability measurement, cells were seeded in 96-well plates at a density of 3000 cells per well in 100 *μ*l medium containing either 25 or 1 mM glucose. When needed, PLD1i or PLD2i was added to the medium at the final concentration of 5 *μ*M. In some experiments, cells were also treated with different concentrations of 2-DG (0.25, 0.5, 1, 2.5, and 5 mM), 20 *μ*M oleic acid (Sigma-Aldrich), 200 *μ*M etomoxir (Sigma-Aldrich), 1 *μ*M methyl-pyruvate (Santa Cruz Biotechnology, Dallas, TX, USA), or 0.1 mM NAC (Sigma-Aldrich). The numbers of viable cells were measured at indicated time points by CyQuant Cell Proliferation Assay Kit (Life Technologies).

### Lentivirus production and transduction

The shRNAs for PLD1 and PLD2 have been described in previous studies.^11, 41, 43^ Lentiviruses were generated by co-transfection of the lentiviral vector (luciferase, PLD1, PLD2, and ATG5 shRNAs in pLKO vector, or pHluorin-mKate2-LC3), pMDLg/pRRE, pRSV-Rev, and pMD2.G in TLA-293T cells (Thermo Fisher, Waltham, MA, USA) using Lipofectamine and Plus reagent (Life Technologies). After infection with lentiviruses, MDA-MB-231 cells expression shRNAs were selected with puromycin before experiments.

### Western blotting

Whole-cell lysates were generated in radioimmunoprecipitation assay (RIPA) buffer containing protease and phosphatase inhibitor cocktails on ice. After brief sonication, protein samples were load on SDS-polyacrylamide gels and transferred onto nitrocellulose membranes that was blocked in 0.1% casein and probed with the indicated primary antibodies. Fluorescently labeled secondary antibodies were used for western blotting and detected by the Li-COR Odyssey infrared imaging system from Li-COR Biotechnology (Lincoln, NE, USA).

### Confocal and fluorescence microscopy

Cells grown on glass coverslips were fixed with 4% paraformaldehyde in phosphate-buffered saline (PBS) for 10 min at room temperature, then permeabilized with 0.1% Triton X-100 or 0.1% Saponin in PBS for 10 min. After blocking with 5% goat serum in PBS, cells were incubated with the desired primary antibody for 1 h at room temperature. Subsequently, cells were washed in PBS and incubated with Alexa 488 or Alexa 594-labeled secondary antibody, or BODIPY493/503 (0.5 *μ*g/ml) (Life Technologies) for 1 h at room temperature. After washing with PBS, the coverslips were mounted on glass slides with 4% n-propyl gallate. Cells were visualized with a Zeiss Axiophot epifluorescence microscope (Carl Zeiss, Thornwood, NY, USA) or a Nikon A1 confocal microscope (Nikon Instruments Inc., Melville, NY, USA). The colocalization and measurement were quantified using ImageJ software (Bethesda, MD, USA).

### Measurement of intracellular ATP and mitochondrial ROS levels and mitochondrial membrane potential

The intracellular ATP levels were measured using the ATPlite Luminescence ATP Detection Assay System (Perkin Elmer, Waltham, MA, USA). The ATP levels of the treated cells were normalized to the mean value obtained from untreated cells. Cytosolic and mitochondrial ROS were detected by flow cytometry after loading the cells with 5 *μ*M DCFH-DA [2-(2,7-dichloro-3,6-diacetyloxy-9H-xanthen-9-yl)-benzoic acid] (Cayman Chemical, Ann Arbor, MI, USA) and 2 *μ*M MitoSOX Red mitochondrial superoxide indicator (Thermo Fisher), respectively. Mitochondrial membrane potential was measured by the fluorescent intensity of TMRE (Tetramethylrhodamine, ethyl ester) (Thermos Fisher) using flow cytometry after staining the cells with 50 nM TMRE for 15 min at 37 °C.

### Determination of lysosomal pH

Cells seeded on 35-mm glass-bottom dishes (MatTek, Ashland, MA, USA) were incubated with 5 mg/ml Oregon Green 488 dextran 10 000 MW (Life Technologies) overnight, followed by 2 h chase in dextran-free medium. At the end of the chase period, the cells were rinsed with and maintained in the imaging buffer (140 mM NaCl, 5 mM KCl, 2 mM CaCl_2_, 1 mM MgSO_4_, 10 mM HEPES, 10 mM Glucose, pH 7.4). Fluorescence images were acquired using an inverted Nikon ECLIPSE TE200 microscope at excitation wavelengths of 440 nm and 490 nm and emission wavelength of 545±50 nm, with a CCD camera controlled by InCytIM-2 software (Intracellular Imaging Inc., Cincinnati, OH, USA). The 490/440 ratio was calculated for each region of interest representing individual lysosomes or lysosomal clusters and converted into a pH value using the pH calibration curve generated from the same coverslip after the initial ratio measurement. To generate the lysosomal pH calibration curve, potassium isotonic solution (10 mM HEPES, 10 mM MES, 140 mM KCl, 1 mM MgCl_2_, 1 mM CaCl_2_, 5 mM glucose) of pH 3.5, 4, 4.5, 5, 5.5, 6, 6.5, and 7.0 containing 10 *μ*g/ml nigericin were sequentially added to cells and each was equilibrated for 10 min before ratio images were taken. Lysosomal pH values were obtained by fitting the obtained intensity ratios to the standard curves.

### Statistical analysis

All statistical analyses were evaluated using unpaired two-sample Student's *t*-test. Data are shown as mean±S.D. *P<0.05; **P<0.01; ***P<0.001.

## Abbreviations

2-DG: 2-deoxy-D-glucose
ATP: adenosine triphosphate
ERK: extracellular signal-regulated kinase
FA: fatty acid
FAO: fatty acid oxidation
NAC: N-acetylcysteine
PA: phosphatidic acid
PBS: phosphate-buffered saline
PLD: phospholipase D
PLD1: phospholipase D1
PLD1i: PLD1 inhibitor
PLD2i: PLD2 inhibitor
shRNA: small hairpin RNA
ROS: reactive oxygen species
tfLC3: tandem monomeric red fluorescent protein (mRFP)
GFP: green fluorescent protein
