# Supplementary material for: Phospholipase D1-regulated autophagy supplies free fatty acids to counter nutrient stress in cancer cells
Source: Cell Death Dis. 2016 Nov 3;7(11):e2448–. doi: 10.1038/cddis.2016.355 (PMC5260880; doi:10.1038/cddis.2016.355)
Supplement: Supplementary Information [file cddis2016355x1.pdf]

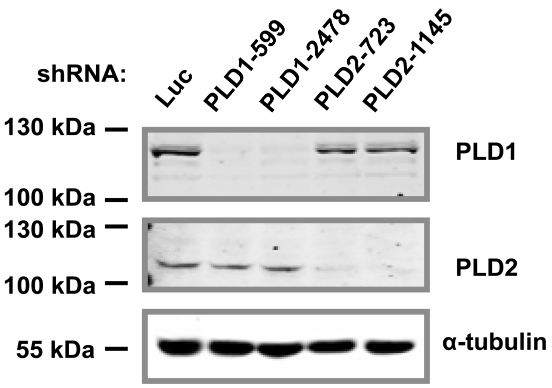

Supplemental Fig S1. Western blot of PLD1 and PLD2 knockdown by two independent shRNAs in MDA-MB-231 cells.

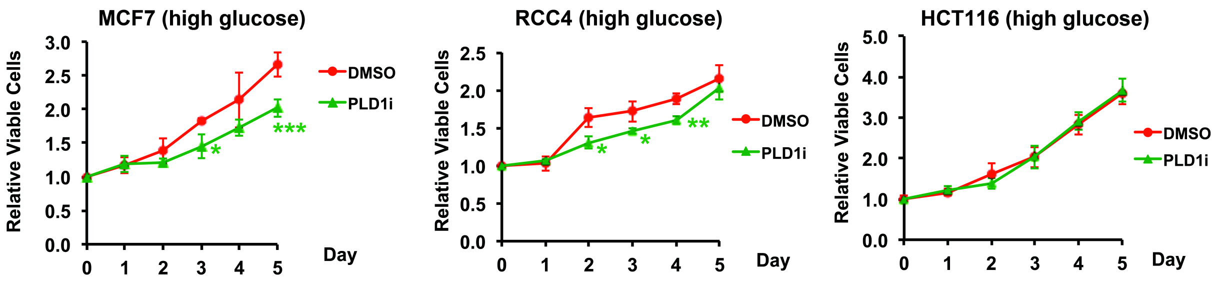

Supplemental Fig S2. PLD1 inhibitor has less effect on the number of viable MCF-7 (breast), RCC4 (renal), and HCT116 (colorectal) cells, in high glucose medium. n=3. Bars show means±SD.

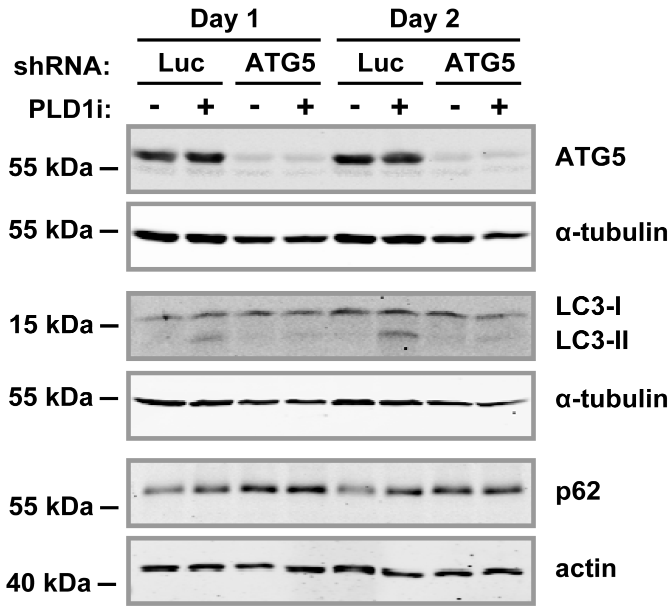

Supplemental Fig S3. ATG5 knockdown abolishes PLD1i-induced increase of LC3-II and p62 levels. MDA-MB-231 cells infected with the control luciferase (Luc) or ATG5 shRNA were cultured in low glucose medium (1mM) in the presence or absence of a PLD1 inhibitor for 1 or 2 days. Total cell lysates were analyzed by Western blotting for the indicated proteins.

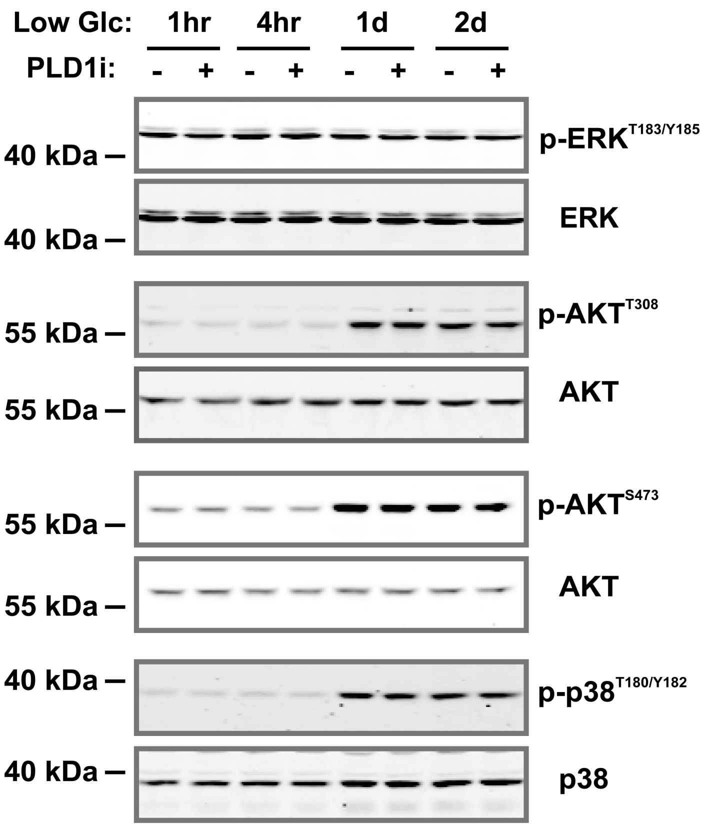

Supplemental Fig S4. PLD1 inhibition does not change the activity of ERK, AKT and p38 signaling pathways. MDA-MB-231 cells cultured in low glucose medium (1mM) were treated with or without a PLD1 inhibitor for the indicated time periods. Total cell lysates were analyzed by Western blotting for the indicated proteins.

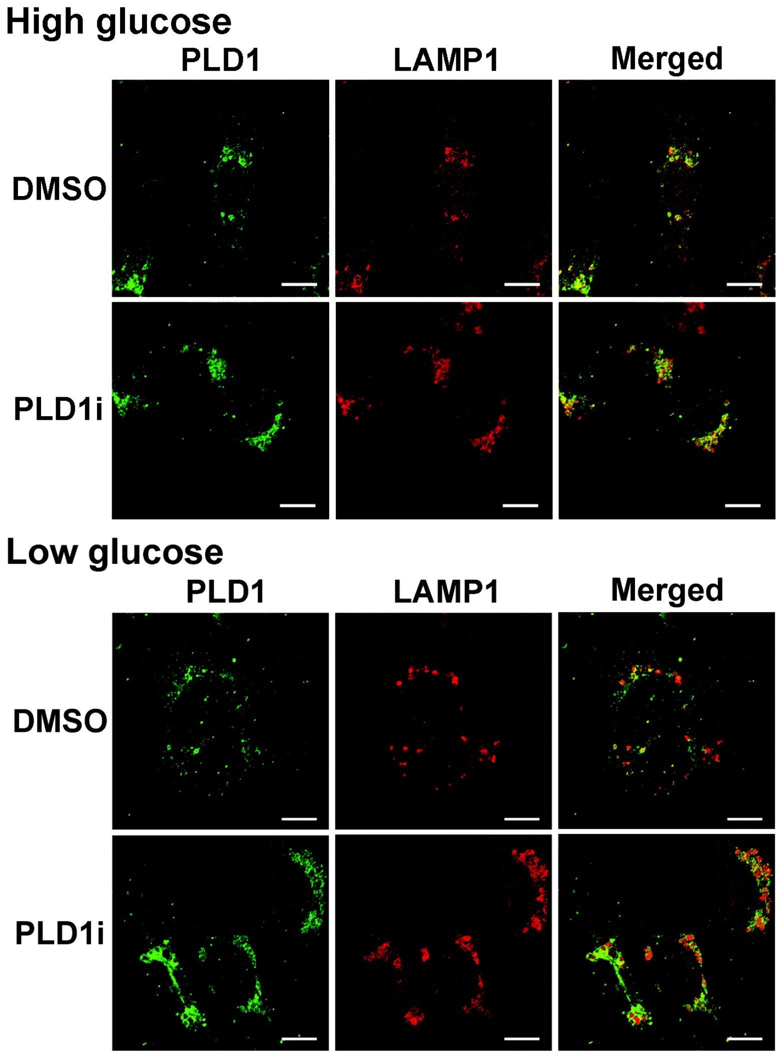

Supplemental Fig S5. A large portion of PLD1 is localized to lysosomes, which is independent of PLD1 inhibitor treatment, in MDA-MB-231 cells grown in both high and low glucose medium for two days (scale bar: 20 μm). Cells were permeabilized with Triton X-100. Lysosomes were labeled by LAMP1.

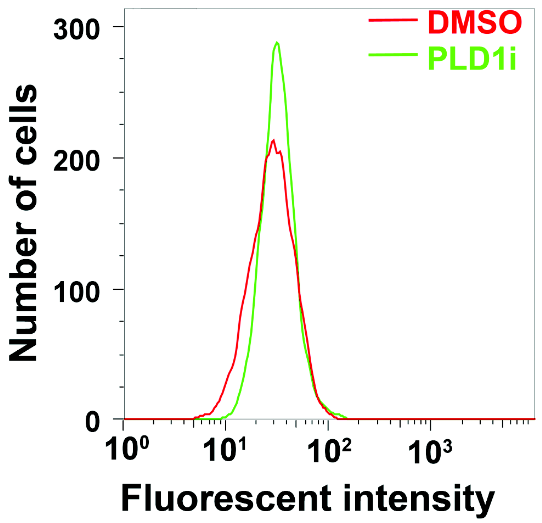

Supplemental Fig S6. PLD1 inhibition does not change the cytosolic ROS level. MDA-MB-231 cells were cultured in low glucose medium (1 mM) in the presence or absence of PLD1 inhibitor for 3 days. Cytosolic ROS were measured by DCFH-DA using flow cytometry.
